# Critical evaluation of the use of artificial data for machine learning based *de novo* peptide identification

**DOI:** 10.1016/j.csbj.2023.04.014

**Published:** 2023-04-17

**Authors:** Kevin McDonnell, Enda Howley, Florence Abram

**Affiliations:** a Functional Environmental Microbiology, School of Natural Sciences, Ryan Institute, University of Galway, Ireland; b School of Computer Science, University of Galway, Ireland

**Keywords:** *de novo*, Artificial data, Synthetic data, Peptide sequencing, Noise

## Abstract

Proteins are essential components of all living cells and so the study of their in situ expression, proteomics, has wide reaching applications. Peptide identification in proteomics typically relies on matching high resolution tandem mass spectra to a protein database but can also be performed *de novo*. While artificial spectra have been successfully incorporated into database search pipelines to increase peptide identification rates, little work has been done to investigate the utility of artificial spectra in the context of *de novo* peptide identification. Here, we perform a critical analysis of the use of artificial data for the training and evaluation of *de novo* peptide identification algorithms. First, we classify the different fragment ion types present in real spectra and then estimate the number of spurious matches using random peptides. We then categorise the different types of noise present in real spectra. Finally, we transfer this knowledge to artificial data and test the performance of a state-of-the-art *de novo* peptide identification algorithm trained using artificial spectra with and without relevant noise addition. Noise supplementation increased artificial training data performance from 30% to 77% of real training data peptide recall. While real data performance was not fully replicated, this work provides the first steps towards an artificial spectrum framework for the training and evaluation of *de novo* peptide identification algorithms. Further enhanced artificial spectra may allow for more in depth analysis of *de novo* algorithms as well as alleviating the reliance on database searches for training data.

## Highlights

• 77% of peaks in spectra are unaccounted for.
• At least 1/3 of matched peaks are random.
• Artificial spectra currently lack complexity of real spectra.
• Modification of artificial spectra increases utility by 156%.

## Graphical Abstract

**Figure:**
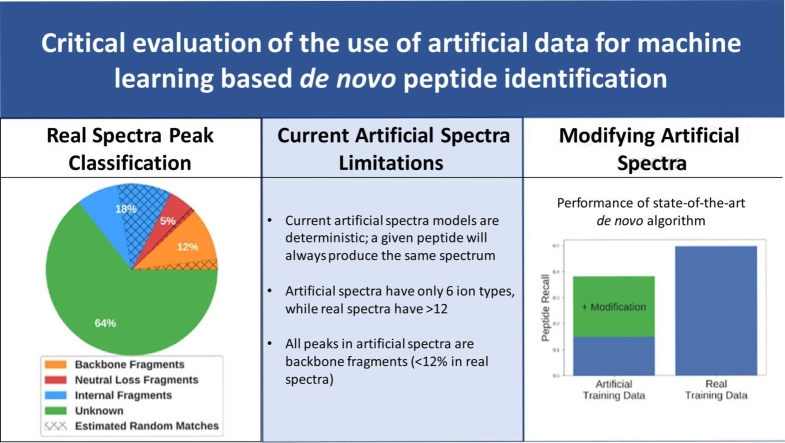

## 1 Introduction

Proteomics can provide valuable insight into the functional profile of a biological system through the identification of the proteins present at the time of sampling [1]. This process is typically performed using a bottom-up strategy, whereby proteins are first digested down in smaller sub-sequences of amino acids called peptides [2]. Peptides are then detected using tandem mass spectrometry (MS) before being mapped back to the corresponding protein. Peptide identification using tandem MS can be accomplished through two main algorithmic approaches; database searching or *de novo* identification [3]. Database searching has been the dominant method for the last few decades, with a higher peptide identification rates than its alternative. In this approach, theoretical spectra are first created for each peptide in the protein database. Each peptide is then given a score based on the similarity between its theoretical spectrum and the observed spectrum. Finally, significant peptide spectrum matches (PSMs) are used to infer proteins identification. Database searching is not without its limitations however. In this approach only 25% of spectra receive significant matches [4]. This is partly because larger database sizes lead to an increased probability of random matches [5]. To limit the number of these false positives, the score threshold for an acceptable PSM must be increased, meaning many correct matches are lost. In this context, *de novo* peptide identification offers a promising database free alternative.

*De novo* peptide identification relies on the spectrum alone to determine the originating peptide sequence [6]. Models are designed to recognise the patterns associated with peptide fragmentation, such as mass differences between ions, to identify amino acids. With an abundance of data now available on repositories such as PRIDE [7], training complex machine learning models for *de novo* peptide identification has become the norm. Indeed, machine learning models are now an integral part of all current state-of-the-art *de novo* peptide identification algorithms [8], [9], [10]. The aim of these models is to learn the fragmentation patterns from the observed spectra that are indicative of the labelled peptide. They are generally trained and tested on real tandem MS spectra, labelled using a database search. However, database searches can be prone to errors meaning the quality of the training data may be suboptimal. Even though target-decoy methods are used to estimate the false discovery rate of database searches, this strategy can still underestimate the number of incorrect matches [11]. This means that *de novo* methods lack ground truth data with which they can be evaluated.

Peptide identification is not straightforward due to the complexity of the peptide fragmentation process. Different fragmentation patterns are observed depending on a multitude of factors such as the amino acid composition of the peptide, the peptide length, the peptide charge and the method of excitation [12]. Between each two amino acids along the peptide chain, there are three different bonds where cleavage can occur. Cleavages at these bonds vary in frequency depending on how energetically favourable they are [12]. For common collision based fragmentation methods such as higher energy collisional dissociation (HCD), cleavage at the amide bond is most common, resulting in b and y ions [13]. Depending on their charge state, fragment ions will be observed at different mass-to-charge ratios (*m/z*). They can also lose neutral molecules of ammonia or water causing a further shift in their observed *m/z*. The b ions are conventionally numbered from N-terminus to C-terminus, with y ions numbered from C-terminus to N-terminus.

The score a PSM receives through a database search is dependent on the number of fragment ions matched [14]. The PSMs that receive the highest scores and are used will therefore be biased toward those with greater numbers of matched ions. This means the training data for *de novo* models, which are labelled using a database search, will tend to have fewer fragmentation cleavages missing. In our previous research missing fragmentation cleavages were found to pose a significant challenge to *de novo* algorithms [15]. While spectra with many missing cleavages will inevitably be more difficult to characterise, a lack of these spectra in the training set will exacerbate the problem.

Artificial data may provide a solution to this issue. In many areas of machine learning artificial data are used to train models where data are scarce, low in diversity or biased [16], [17], [18]. This allows for full control over the creation of the data, and therefore over what a model learns from. Artificial data are created such that they match the statistical properties of real data and in doing so, capture the patterns present in the real data [19]. Shmelkov *et al.* developed a method to evaluate the utility of artificially generated data [20]. They trained a classification model on synthetic data created using a generative adversarial network (GAN) and compared the performance to a model trained on real data. Artificial training data that could best replicate the performance of the real data and could therefore replicate the inherent relationships were deemed to be the most useful. Similar methods have been employed by other groups to measure artificial data quality [18], [21], [22].

Artificial data can also be used for evaluation purposes [23], [24]. For example, artificial test data can be designed to contain scenarios which are unlikely to appear in a real dataset. This is extremely useful to test a system against important but rare events [25]. Furthermore, the flexibility of artificial data means the performance of models can be tested across a wide range of scenarios.

In the context of peptide identification, there are many models available to create artificial peptide spectra [26], [27], [28]. Prosit is a state-of-the-art, open-source, spectrum prediction model [29]. It uses machine learning to capture the relationship between fragment ion intensity and peptide sequence, producing high quality artificial spectra. The model is capable of predicting the b and y ion fragment peaks of peptides up to length 30. While the theoretical spectra traditionally used in a database search only contain the *m/z* location and an arbitrary intensity, Prosit’s spectra are highly correlated to the observed fragmentation pattern. The authors successfully used this additional information to increase the number of identified peptides by up to 35% compared to the *m/z* alone [29].

To advance the field of *de novo* peptide identification, a greater understanding of both the strengths and limitations of current algorithms is required. Artificial test data would facilitate this by providing spectra with specific characteristics allowing researchers to understand how their algorithms perform for varying levels of complexity and noise. Artificial data have previously been used to evaluate peptide identification models but only to a limited degree and are generally used as a secondary analysis with unvalidated additions of noise [15], [30], [31]. While artificial spectra have proven useful for database identification, their relevance to *de novo* identification has never been addressed. Furthermore, using artificial spectra as training data for *de novo* models may help circumvent the current bias associated with database labelling. To address the knowledge gaps, we evaluate the utility of artificial data in the context of *de novo* peptide identification. We first analyse real data and categorise the different forms of noise which can be present. We also estimate the rate of spurious ion matches in the mass spectra using random non-matching amino acid sequences. Then, through the addition of noise, we modify artificial spectra to increase its similarity to real spectra. Finally, we assess the utility of the modified artificial spectra by using them to train the state-of-the-art *de novo* peptide sequencing model PointNovo [9], and compare the difference in performance to the model trained on real spectra.

## 2 Methods

### 2.1 Real spectra

The real spectra used in this research come from 9 different organisms and 9 different research groups (Table 1) [32], [33], [34], [35], [36], [37], [38], [39], [40]. All experiments were conducted with a Thermo Scientific Q-Exactive mass spectrometer by the respective research groups. The raw data were combined and processed by Tran *et al.* [8] using the PEAKS DB software [41] with their respective proteome database. The data were then filtered using a 1% false discovery rate threshold. More information on the data processing and experiments can be found in the original papers [8], [32], [33], [34], [35], [36], [37], [38], [39], [40]. As processing of the MS/MS spectra was carried out prior to this research, the effect of this on the subsequent analysis is not considered.

**Table 1:** Details of nine real datasets used. Accession indicates the PRIDE accession number. FragTol indicates the error tolerance for fragment ions used by Tran *et al.* in the database search [8].

Dataset	Organism	Accession	FragTol (Da)	Reference
Yeast	*Saccharomyces cerevisiae*	PXD003868	0.05	Seidel *et al.*[38]
Human	*Homo sapiens*	PXD004424	0.02	Cypryk *et al.*[40]
Mouse	*Mus musculus*	PXD004948	0.05	Nevo *et al.*[33]
Bacillus	*Bacillus subtilis*	PXD004565	0.05	Reuß *et al.*[35]
ClamBacteria	*Candidatus Thiodiazotropha endoloripes*	PXD004536	0.05	Peterson *et al.*[36]
Honeybee	*Apis mellifera*	PXD004467	0.05	Hu *et al.*[39]
Ricebean	*Vigna mungo*	PXD005025	0.05	Paiva *et al.*[32]
Tomato	*Solanum lycopersicum*	PXD004947	0.05	Mata *et al.*[37]
M. mazei	*Methanosarcina mazei*	PXD004325	0.05	Cassidy *et al.*[34]

The spectra were also partitioned into different datasets by Tran *et al.* [8] to create 9 separate training, validation and testing sets, one for each organism. Each test set consists of spectra from the respective organism while the training and validation sets for each organism are composed of spectra from the other 8. The resulting MGF files were downloaded from ftp://massive.ucsd.edu/MSV000081382/.

In this research, the characteristics of real data were derived from a subsample of 50,000 spectra from the nine organisms, while model training was conducted on the entire yeast partition. These choices were made to limit computational resources while still addressing our research aims. The yeast partition was selected as *Saccharomyces cerevisiae* is a model organism and well characterised.

To allow for a meaningful comparison, the real data were then filtered to exclude spectra which could not be replicated by Prosit. The Prosit pipeline considers carbamidomethylation of cysteine as a fixed modification and oxidation of methionine as the only variable modification. It also cannot predict spectra for peptides with more than 30 amino acids. Hence, PSMs in the datasets longer than this or containing other modifications were removed prior to analysis.

### 2.2 Artificial spectra

Artificial spectra in this experiment were created using the Prosit pipeline [29]. The source code was downloaded from https://github.com/kusterlab/prosit. A pretrained model was downloaded from https://figshare.com/projects/Prosit/35582. The precursor charges and peptide sequences of the spectra were extracted from each dataset. These were then used by Prosit to create an artificial copy of the real datasets used in this study (Table 1).

### 2.3 Peak matching

Theoretical fragment ions were created for each of the database assigned peptides using the Pyteomics Python module [42]. These were then compared to the observed peaks in the spectra collated and processed by Tran *et al.* [8]. Observed peaks that fell within 0.05 Da of a theoretical peak were considered matched (maximum from Table 1). If multiple observed peaks satisfied this condition, the peak with the smallest mass difference from the theoretical ion was considered to be the correct match.

Fragment ions can occur from single bond cleavages along the backbone of the peptide. In this research, three such backbone ion types were considered (a, b and y) and are referred to as backbone ions for the remainder of this manuscript. These ions can also lose neutral molecules of NH_3_ and H_2_O which we refer to as neutral losses. Therefore we consider twelve ion types in total, namely a ions, b ions, y ions, a-H_2_O ions, b-H_2_O ions, y-H_2_O ions, a-NH_3_ ions, b-NH_3_ ions, y-NH_3_ ions, a(2+) ions, b(2+) ions and y(2+) ions. Loss of H_2_O was only considered for C-terminus ions as well as others containing aspartic acid, glutamic acid, serine or threonine [12], [43]. Loss of NH_3_ was only considered for fragments containing the amino acids arginine, lysine, glutamine or asparagine [12], [43].

Internal fragments were also created for each peptide. These are defined as the amino acid sequence arising from two backbone cleavages and can occur in two possible types; a and b [44]. To this end, all k-mers from the second amino acid to the second last were identified and the sum of their masses calculated. The mass of a hydrogen atom was added to give the mass of b-type internal fragments [45]. These masses were then duplicated with the combined mass of a carbon and oxygen removed to create the set of a-type internal fragments.

### 2.4 Random peptides

The number of peaks matched in the spectra that may have occurred by chance was estimated by creating a random peptide for each spectrum. Peptides of the same length were generated by randomly sampling amino acids with probabilities proportional to their prevalence in the set of assigned peptides used in this study (data collated by Tran *et al.* [8]). To generate tryptic peptides the final amino acid in each sequence was set to arginine or lysine, alternative to the last amino acid of the database assigned peptide. This method was used to ensure as little overlap in fragment ion masses as possible while generating random tryptic peptides with the same distribution of amino acids as those observed in real data. Alternative methods explored can be found in Supplementary with results in Supplementary Table 1. Theoretical backbone ions and internal fragments were then created for each random peptide, as was done with the real peptides (see section Peak Matching). These were then matched to the spectra with a tolerance of 0.05 Da.

### 2.5 Data modification

During this research both real and artificial spectra were modified. Four different spectrum features that can contain noise or variability in their observation were identified; *m/z*, intensity, presence/absence of fragment ions and unknown peaks. The effect of the addition and removal of each of these noise types was analysed.

In real data, removal of *m/z* jitter was performed by resetting each peak to its expected value. To do this the *m/z* value for each theoretical ion of the assigned peptide was calculated using the Pyteomics package [42]. The *m/z* of the closest matched peak was then assigned the theoretical *m/z* value (see section Peak Matching).

The *m/z* jitter was reintroduced using two different methods, each approximating the real noise distribution. Method one consisted of a mixture distribution of two normal distributions, both with means of 0 and standard deviations of 1e-2 and 1e-3 respectively in a 1:1 ratio. Method two consisted of a mixture distribution of a Laplace distribution with a mean of 0 and a scale parameter of 2.5e-3 as well as a uniform distribution between − 0.05 and 0.05 with a 12:1 ratio. Jitter in the *m/z* values was introduced to peaks by taking random samples from the respective distributions and adding them to the expected theoretical *m/z*.

When real intensity was modified it was replaced by the Prosit predicted value. However, Prosit did not predict an intensity for all peaks matched in the real data. If a fragment ion was considered unlikely enough Prosit would not create a corresponding peak. If this occurred and the peak was matched in the real spectrum the intensity was left unchanged.

With peaks ordered by intensity, we found there was a linear relationship between the number of peaks to be removed from the Prosit spectra to match the number of missing fragmentation cleavages in real spectra and the peptide length. Using a linear regression model we could approximate the mean number to remove by length using the formula *n* = *max*(*l* − 5, 0), where *n* is the number of peaks removed and *l* is the length of the peptide. Peaks were removed with the lowest intensity first as these were the least abundant ions and therefore most likely to be missing.

Unknown peaks were introduced using three different methods. These methods introduced peaks as singly charged ions as these account for most of the observed peaks. The first method involved the creation of random combinations of amino acids and calculating the sum of their masses. These were introduced as singly charged peaks with their *m/z* value equal to their mass. The second method involved the creation of internal fragments from the peptide assigned to the spectrum. For each peptide, all possible internal fragments of type a and b were created (see section Peak Matching). This created a population of *m/z* values equal to their masses as these were also only considered as singly charged peaks. The third method introduced peaks as a combination of the above methods. For each method, the number of peaks introduced to each spectrum was set equal to the number observed in the equivalent real spectrum for a fair comparison. Unknown peaks were then introduced by randomly sampling from the respective set of created peaks. In the case of the combined method, the number of internal fragments was defined to match their observed occurrence while random combinations of amino acids made up the remaining amount. Intensity values for artificial non-peptide peaks were sampled from a log-normal distribution estimated from unknown peaks in real data in our previous research [46]. The natural log of this distribution has a mean of − 4.4 and a standard deviation of 1.5.

### 2.6 PointNovo

PointNovo is the current state-of-the-art in *de novo* peptide identification [9]. It was used to evaluate the utility of modified and unmodified spectra for training *de novo* models. PointNovo is the updated version of DeepNovo [8] and was previously released as DeepNovoV2 [47]. The source code was downloaded from https://github.com/volpato30/DeepNovoV2. Models were trained on the Yeast partition of the labelled data collected by Tran *et al.*. In this partition the test data come from *Saccharomyces cerevisiae* while the training and validation data come from the other 8 organisms. The models were trained with validation testing occurring every 300 steps as per the original code and the parameters were saved for the lowest validation loss.

### 2.7 Metrics

The metrics used to evaluate the trained models are those used by PointNovo [9]. Firstly, the predicted amino acid and the actual amino are required to have a mass difference of less than 0.1 Da. If this condition is met and the difference between the combined mass of the previously predicted amino acids and the combined mass of the previous actual amino acids is less than 0.5 Da, then an amino acid is considered matched. Amino acid precision is then defined as the total number of matched amino acids over the total number predicted. Amino acid recall is the total number of matched amino acids over the total number of actual amino acids. Similarly, peptide recall is the total number of correct peptides over the total number of spectra.

## 3 Results

A comparison between the performance of a model trained using real and artificial data can be used as an indicator of the quality of the latter [20]. Table 2 shows the performance of PointNovo given three different training and test set combinations of real and artificial spectra. The artificial data are duplicates of the real data, generated using Prosit [29]. The performance of the model when trained and tested on real spectra is the baseline for this research. It provides a reference with which to compare to the model performance when trained using artificial spectra. If artificial training data can reproduce the test performance of real training data it can be considered an adequate replacement [21]. It should be noted that the performance reported here using real training data differs slightly from that reported in the original paper [9] as the real data used in this experiment has been filtered to match the capabilities of Prosit (see Section Real Spectra).

**Table 2:** Performance of PointNovo [9] on real and artificial spectra. The real spectra are from the yeast partition dataset collated by Tran *et al.*[8]. The artificial spectra are from a duplicate dataset created using Prosit [29]. Test data are composed of *Saccharomyces cerevisiae* spectra with training data made up of spectra from 8 other organisms. AA stands for amino acid.

Train Data	Test Data	AA Recall	AA Precision	Peptide Recall
Real Spectra	Real Spectra	0.7160	0.7158	0.4971
Prosit Spectra	Real Spectra	0.3764	0.3743	0.1487
Real Spectra	Prosit Spectra	0.9277	0.9286	0.7238

When PointNovo was trained on artificial spectra created using Prosit, its test performance on real data dropped dramatically (Table 2). Peptide recall fell by 70% with amino acid recall and precision falling by 47% and 48% respectively (Table 2). In contrast, a model trained on real data and tested on artificial data appears to perform much better than the baseline of real test data. For the artificial test set created using Prosit, peptide recall was 46% greater than the real data test performance with both amino acid recall and precision both increased by 30% (Table 2). These results indicate that current artificial spectra models are not an adequate representation of tandem mass spectra for *de novo* evaluation. While they may provide accurate predictions of fragment ions, they lack the noise and random variation associated with real spectra. Artificial spectra provide a much simpler representation for the model to learn which is highlighted by the reduced performance when used for training, and the increased performance when used for testing. We therefore examined the distinctive characteristics of real spectra to appropriately modify artificial spectra.

### 3.1 Classification of peaks

Due to the complexity of tandem mass spectra resulting from peptide fragmentation, many algorithms and models only consider backbone ions attributable to the peptide. Likewise, artificial spectra prediction algorithms, such as Prosit, only train their models to predict b and y fragment ions [29]. While these ions are important as they can reveal the peptide, they make up only a fraction of the total number of peaks [15]. Little work has been done to classify the other peaks in the spectra in the context of *de novo* peptide identification, despite their overwhelming majority. Here we aim to classify as many peaks as possible using the data collated by Tran *et al.* [8].

Not all possible peptide fragments will appear in the matched spectra. This is because the creation of some fragments will be more energetically favourable than others [12]. Table 3 shows the numbers of fragment ions matched in a sample of 50,000 spectra. This sample size was used to limit computational resources. The number of possible ions were calculated for each ion type using the peptides assigned during the database search conducted by Tran *et al.* [8].

**Table 3:** The number of matched peaks of different ion types in a sample of 50,000 HCD PSMs with a matching tolerance of 0.05 Da. The data are from 9 different organisms and research groups, collated by Tran *et al.*[8]. Columns indicate the number of possible ions of each type from the assigned peptides (#Possible), the number of these possible ions that were matched in the spectra (#Matched), the fraction of the possible ions that were matched (Fraction Matched), the number of ions from random peptides that were matched (#Random), and the ratio of the number of ions matched from the random peptides to the number of ions matched from the assigned peptides (#Random/#Matched).

Ion Type	#Possible	#Matched	Fraction matched	#Random	#Random #Matched
Backbone	1934880	929072	48%	117596	13%
a	644960	154436	24%	35959	23%
b	644960	272490	42%	29728	11%
y	644960	502146	78%	51909	10%
Charge 2+	1934880	160515	8%	78457	49%
a(2+)	644960	43336	7%	19616	45%
b(2+)	644960	50799	8%	16811	33%
y(2+)	644960	66380	10%	21224	32%
Ion Loss	1842317	497932	27%	57651	12%
a-H20	143726	26641	19%	7138	27%
b-H20	143726	51607	36%	5050	10%
y-H20	644960	216682	34%	43334	20%
a-NH3	303529	39709	13%	8372	21%
b-NH3	303529	65583	22%	6093	9%
y-NH3	302847	97710	32%	8471	9%
Int. Frag.	7755678	1661562	21%	934571	56%
b	3877839	1031737	27%	474481	46%
a	3877839	629825	16%	460090	73%

As with any matching task there is a probability that some of the matches will occur by chance through the random alignment of a non-fragment peak with the position of an expected fragment ion peak. To estimate the occurrence of this phenomenon, a random peptide was created for each spectrum and all matching fragment ions identified. The most abundant ions were b and y backbone ions as expected (Table 3) [13]. They accounted for approximately 3% and 6% of the total peaks in the spectra respectively. Both of these ion types also matched the largest fraction of their possible peaks. 42% of all possible b ions were matched as well as 78% of all possible y ions (Table 3). Of the b and y ion matches observed, we estimate the fraction of random matches to be 11% and 10% respectively. The other backbone ion type analysed, a ions, were matched in much smaller numbers accounting for 1.8% of all peaks as they only matched 24% of those possible (Table 3). The estimate for the fraction of matches which occurred randomly was also higher for a ions at 23%.

**Fig. 1:**
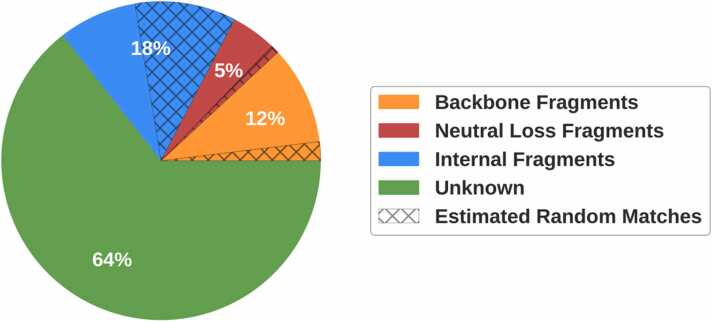
Fraction of peaks accounted for a sample of 50,000 HCD spectra. Percentages indicate the fraction of the total number of peaks each segment represents. Hatching indicates the proportion of each ion type estimated to have been matched by chance. The data are from 9 different organisms and research groups, collated by Tran *et al.*[8].

Fragment ions attributable to the database assigned peptide make up only a fraction of the peaks in MS/MS spectra (Fig. 1). Backbone ions (a,b,y), both singly and doubly charged, were found to account for approximately 12% of all the peaks in the spectra. Of this 12%, 18% were estimated to be random (2% of the total). The fraction of peaks accounted for by backbone ions is almost half of what was estimated in previous work for HCD spectra from an LTQ Orbitrap Velos mass spectrometer [48]. Similarly, fewer matches were also observed for neutral loss ions in our experiment. Backbone ions with a neutral loss of a water or ammonia molecule accounted for just 5% of the total (Fig. 1).

A substantial proportion of the peaks in the spectra could be attributable to internal fragments (18%, Fig. 1). This is in part due to the large number of possibilities for this ion type; for a peptide of length 10 with 10 unique amino acids, there are 28 possible unique internal fragments of each ion type. This is compared to 9 possible backbone fragments of each ion type for the same peptide length. Also, the number of possible internal fragments grows exponentially for longer peptides. Furthermore, we consider two ion types (a and b) for each internal fragment which will double the number of possible peaks [45], [49]. Table 3 shows that 21% of the possible internal fragments were matched to a peak in the spectra. However, many of the internal fragments from the random peptides were also matched in the spectra. By comparing the number of internal fragments matched for both the assigned and random peptides, 49% of the actual internal fragment matches were estimated to have occurred by chance (Fig. 1).

### 3.2 Distribution of *m/z* error

**Fig. 2:**
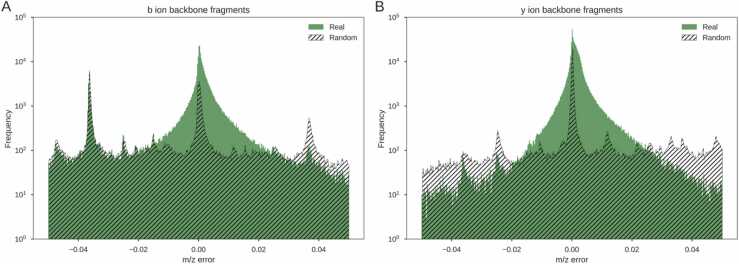
Distribution of error in matched peak *m/z* for singly charged b and y ions from a sample of 50,000 HCD spectra. The data are from 9 different organisms and research groups, collated by Tran *et al.*[8]. A shows the error distribution of matched b ions. B shows the error distribution of matched y ions. Error for ions from the real peptides are shown in green, with errors from the random peptides in black hatching.

The observed *m/z* values of peptide fragment ions may differ slightly from their theoretical values. These errors may be random or systematic [50]. Systematic errors are caused by biased measurements that result in repeatedly observed errors. To quantify the measurement error of the matched peaks, the mass difference between each matched ion and its expected value was recorded. A mass tolerance of 0.05 Da was used, so each peak matched fell within this error range [15]. Fig. 2 shows the distribution of the difference in mass between the observed and theoretical values for singly charged, b and y ions. It also shows the error distribution for the peaks matched to the randomly generated peptides. In general the real peptide jitter is centered around zero indicating random measurement error.

There is a considerable difference between the error distributions of matched peaks from real and random peptides (Fig. 2). It should be noted figure uses a log scale on the y axis. The b ions from real peptides had a mean squared error over 4 times lower than the random peptides (1.6e^−4^ vs 7.1e^−4^). The y ions from the real peptides had a mean squared error of 3.4^−5^ with random peptides over 9 times larger at 3.2^−4^. Notably, the random distribution matches the real distribution at the tails, especially for the b ions. This suggests that many of the real matches have occurred by chance. A similar method to estimate the number of spurious matches has been used by Goloborodko *et al.* [51]. Without using random peptides, they assumed the spurious matches formed a uniform distribution over the entire window below which the frequency never fell. Our random peptide matches show a similar distribution with a constant rate across the whole window, validating their approach.

The random distribution of y ions in Fig. 2B shows a very large spike at 0.00 *m/z* error. This is partially attributable to the prevalence of y_1_ ions of arginine and lysine present in most spectra (Supplementary Table 2). The random distribution in Fig. 2B also has higher tails compared to the distribution of real y ions. However, we were unable to account for this difference.

For both plots in Fig. 2, multiple significant secondary peaks in the error distribution can be observed along the x-axis, in addition to the peak at zero. These are particularly pronounced in Fig. 2A showing the b ions. The two largest secondary peaks in the distribution occur around 0.036 Da each side of the origin. This is approximately the mass difference between lysine (K) and glutamine (Q) which may explain this phenomenon. While they share most of their constituent atoms, lysine has an additional CH_4_ while glutamine has an additional oxygen. Actual spectrum peaks and the possible fragment peaks that share a similar difference in chemical composition and consequently mass, will therefore produce the observed secondary peak in the distribution.

### 3.3 Abundance of different ion types

Missing peaks, and hence missing fragmentation cleavages were found to be the greatest challenge *de novo* peptide identification algorithms must overcome [15]. Missing peaks occur when the abundance of a fragment ion, which is represented by peak intensity, is below the detection limit. The abundance of a fragment ion is dependent on how energetically favourable the corresponding cleavage is, which in turn depends on the cleavage position, peptide sequence and method of fragmentation [12], [52].

**Fig. 3:**
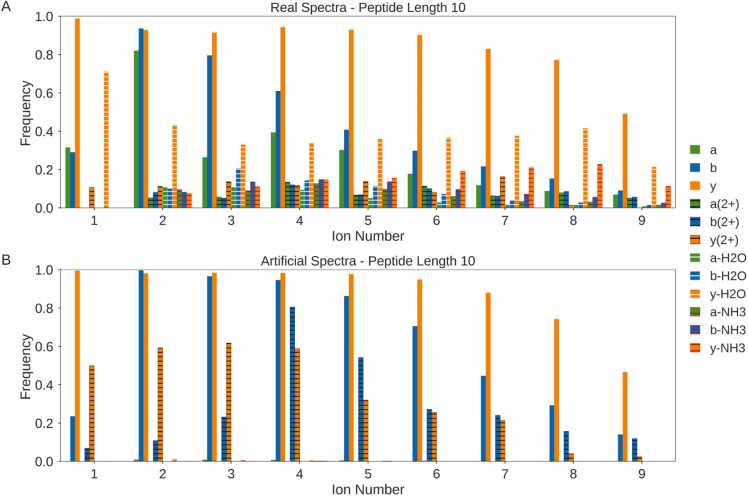
Comparison of the distribution of 12 different ion types in real versus artificial spectra for length 10 peptides in a sample of 50,000 HCD spectra. Frequency denotes the fraction of spectra where each ion was present. The real data (A) are from 9 different organisms and research groups, collated by Tran *et al.*[8]. The artificial spectra (B) are from a duplicate dataset created using Prosit [29]. Ions of the same type share the same base colour with different colour hatching indicating different charge states or neutral losses.

Figure 3 shows a comparison of the distribution of the presence and absence of fragment ions from 12 different ion types in both real and artificial spectra with assigned peptides of length 10 (median length of the data used).

On average, the artificial data were found to have more fragment ions present than the real data for the ion types that are predicted by Prosit [29]; b and y ions, both singly and doubly charged. In particular, b ions are much more prevalent in the artificial spectra. For y ions, low ion numbers are consistently more prevalent in artificial spectra while some higher ion numbers are more prevalent in real spectra (Fig. 3). Both of these differences contribute to the observation that artificial spectra have fewer missing cleavages than real spectra [15], as at least one fragment ion is present for most cleavage sites. The reason for the increased number of fragments in artificial spectra may be partly due to the fact that artificial data do not contain measurement error or background noise meaning low intensity fragment ions are not lost. Hence, each ion predicted by Prosit will be present in the artificial spectra.

The overall trends for the b and y ion series share some similarities between the real and artificial data (Fig. 3). For both data types in the b ion series, the b_1_ ion has a low frequency before a large increase to the frequency of the b_2_ ion. The frequency of the b ions then decreases for increasing ion numbers. Also, both data types show a generally decreasing frequency in the y ion series with the y_1_ ion as the most frequently observed and the y_9_ ion as the least frequently observed.

The difference in the number of missing peaks between artificial and real data was found to be related to the length of the peptide. A greater deviation in ion distributions was observed between real and artificial spectra for longer peptides (Supplementary Figures 1–3).

### 3.4 Differences in peak intensity

Prosit produces extremely accurate fragment ion intensity predictions with a reported median spectral angle of 0.92 [29]. It uses a deep learning model composed of multiple recurrent neural network layers and fully connected layers to encode the peptide sequence and make the prediction. However, the Prosit model is deterministic and so for a particular amino acid sequence, it will produce the exact same spectrum every time. This is not the case in real spectra where variability is typically observed between spectra of the same peptide [53].

We compared the intensity predictions of Prosit to those observed in the real spectra for peptides of length 10 (Supplementary Figure 4) by determining the distribution of differences in intensity between Prosit and the real spectra. Only b and y ions both singly and doubly charged were used as these are the only frequently observed ion types predicted by Prosit. All intensities in real data were normalised to the maximum intensity of the fragment ions and not the spectrum, to provide a fair comparison with Prosit. However, in real data, a fragment ion may be the most intense peak in approximately half of the spectra [15]. The median difference between the artificial and real intensity values was less than 0.05 for all ion types from length 10 peptides, indicating a very low prediction error. Similar trends were observed for other length peptides (Supplementary Figures 5–7). This evaluation confirms the high accuracy reported by Prosit in the original manuscript [29].

### 3.5 Quantifying internal fragments

Internal fragments are caused by the cleavage at two or more backbone bonds in a peptide [12]. This results in fragments whose amino acids are not a sequence beginning at one end of the peptide, but instead are an internal sequence. Internal fragments have not yet been utilised in *de novo* peptide identification algorithms as their inclusion was found to make algorithms prohibitively complex [54]. This is despite their prevalence in tandem mass spectra as shown in Fig. 1. Here we analyse which internal fragments are observed in real spectra and which may have been matched by chance.

**Fig. 4:**
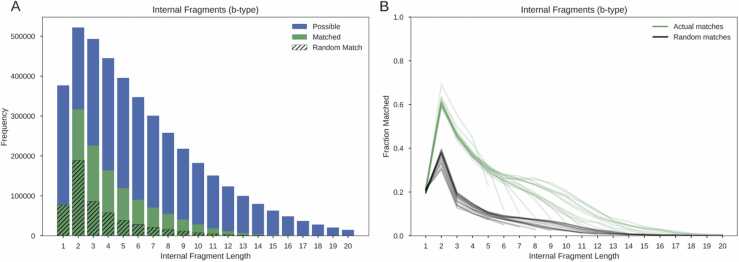
The number of b-type internal fragments matched by length in a sample of 50,000 HCD spectra. The data are from 9 different organisms and research groups, collated by Tran *et al.*[8]. A shows the counts of possible unique internal fragment masses (blue), matched internal masses (green), matched random internal masses (black hatch). B shows the fraction of the total number of possible internal fragments matched by the actual peptides (green) and the random peptides (black). Each individual line represents a different peptide length.

Figure 4A shows the frequency of occurrence of different length b-type internal fragments in real spectra, and the estimated frequency of randomly matched internal fragments using random peptides. Internal fragments of length two had the greatest frequency of all internal fragment lengths for both the actual and random peptides. The fraction of possible unique length two internal fragments matched by the actual peptides (60%) was also greater than any other internal fragment length for all peptide lengths (Fig. 4B).

Notably, the fraction of b-type internal fragments of length one that were matched for the assigned peptides (21%) was almost exactly the same as the fraction of length one fragments that were matched for the random peptides (21%). This trend was also observed for a-type internal fragments (Supplementary Figure 8).

### 3.6 Identification of unknown peaks

Most of the peaks in tandem MS spectra come from ions of unknown origin (Fig. 1). These peaks are generally referred to as noise which can be chemical or electrical in nature [55]. The molecular structure of these ions can be used to investigate their origin. As molecules are made of atoms, most of their mass comes from protons and neutrons, together known as nucleons. The dalton (Da) is defined as 1/12th the mass of a carbon-12 atom, the average mass of one of its nucleons. Therefore, if chemical noise is present, it should appear at roughly integer multiples of the dalton while electrical noise will not. Very few peaks were found to fit the criteria for electrical noise and instead almost all appeared to cluster at approximately integer multiples as expected for chemical noise (Supplementary Figure 9A). However, the clusters drifted off the integer units for larger *m/z* values (Supplementary Figure 9B).

**Fig. 5:**
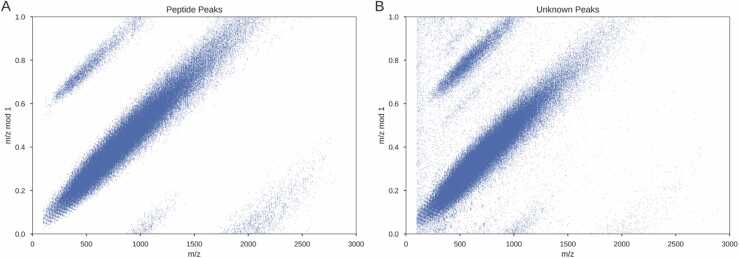
Distribution of *m/z* values vs *m/z* modulo 1 for peptide fragment peaks and unknown peaks in a sample of 50,000 HCD spectra. The data are from 9 different organisms and research groups, collated by Tran *et al.*[8]. A shows the distribution of the *m/z* values from peaks attributable to the database assigned peptide. B shows the distribution of the *m/z* from all other peaks.

To investigate the nature of this phenomenon in tandem mass spectra from shotgun proteomics we looked at the distribution of *m/z* values when plotted against the *m/z* modulo 1 (Fig. 5). The modulo operation shows the remainder of the division after the modulus (in this case 1) has been divided in evenly.

The distribution of *m/z* versus *m/z* modulo 1 shows clear patterns for both the matched and unmatched peaks (Fig. 5). There are two clear streaks in Fig. 5A that wrap around from the top of the plot to the bottom. The wrapping is caused by the modulo operator. If the mass of these ions were integer multiples of the dalton, there would be a horizontal line across the plot. However, the streaks appear at a slope of approximately 1.0005. While this is only a slight deviation, it is consistent meaning larger ions appear significantly far from the integer values. This value agrees with previous reports of the average distance between peaks in tandem mass spectra [56], [57].

The binding energy of atoms can differ depending on the make-up of their nucleus. This means their masses can deviate from these integer values of the dalton. The dalton is normalised to the binding energy of carbon. With different binding energies for different atoms, almost all areas of the plot could be reached using different combinations of atomic masses. However, most of these atoms are extremely rare so instead we checked if these unknown peaks come from atoms common in biological molecules; i.e. molecules composed mostly of hydrogen, carbon, nitrogen, oxygen and sulphur. Supplementary Figure 10 is an *m/z* vs *m/z* modulo 1 plot showing random masses using equal ratios of these atoms, peptide ratios of these atoms, and carbohydrate ratios of these atoms using the formula C_*n*_(H_2_O)_*x*_ [58]. The relative ratios of hydrogen, carbon, nitrogen, oxygen and sulphur in peptides were calculated using the Pyteomics module [42]. The streak shown in the real data matches the distribution of the peptide ratio (Supplementary Figure 10). This would suggest that the noise observed in the real data is composed of peptide-like fragments. Calculating the average mass per nucleon given the ratio of these elements observed in peptides equates to 1.0005, equal to the slope we calculated earlier.

The parallel streaks observed in these distributions are caused by different charges (Supplementary Figure 11). As only two charge types are considered for the peptide ions in Fig. 5A, only two streaks are found. Conversely, there are more streaks in Fig. 5B showing the presence of (3+) ions. Fig. 5B has also a background level of noise which does not fit into any of the streaks which may be electrical in nature.

Finally we check to see if the observed streaks could also be obtained by contaminant metabolites. The human metabolome was downloaded from https://hmdb.ca/downloads[59]. Supplementary Figure 12 shows the ions of these metabolites for different charges. While there is an overlap between the metabolites and our observed peaks, the pattern is clearly different. While peptides have a limited set of possible masses corresponding to combinations of the 20 amino acids, metabolites have much greater variety leading to the wider observed streaks. Again, this suggests that the unknown peaks are of peptide origin, albeit not the assigned peptide.

### 3.7 Evaluation of modified artificial training data

We then aimed to improve the similarity between Prosit generated spectra and real spectra through the addition of artificial noise. First real training data were modified to observe the effect of each noise type on PointNovo performance [9]. Table 4 shows the performance of the model when the different types of noise and variability, not present in artificial data, are removed from the real training data.

**Table 4:** Performance of PointNovo [9] when trained using modified real spectra. The training data had noise removed from the four different spectrum attributes separately. The data are from the yeast partition dataset, collated by Tran *et al.*[8]. Test data are composed of *Saccharomyces cerevisiae* spectra with training data made up of spectra from 8 other organisms.

Noise Type Removed	AA Recall	AA Precision	Peptide Recall
*m/z* Jitter	0.6503	0.6497	0.4274
Intensity Variation	0.7150	0.7149	0.4916
Missing Peaks	0.6578	0.6547	0.4436
Non-Backbone Peaks	0.4957	0.4872	0.2452

The removal of variation associated with the intensity of a peptide peak had the least effect with peptide recall decreasing by just 1% (Table 4). For this analysis, intensity values for the matched fragment ions were replaced with the Prosit equivalent. The small reduction indicates the high quality of the predicted intensity, agreeing with our earlier analysis (Supplementary Figures 4–7). As the reduction in performance was so small we did not try to modify the intensity further.

Artificial spectra generated by Prosit only contain backbone ions [29]. The removal of non-backbone peaks from real spectra, leaving only these backbone ions, resulted in a decrease in peptide recall of 51% (Table 4). These peaks account for over two thirds of the total number of peaks in tandem MS spectra (Fig. 1). Without them, the model only needs to learn a mapping from the backbone peaks to the peptide, making the task much simpler. The model then overfits the data leading to reduced test performance. This supports the results observed for artificial training data (Table 2).

To test if the pattern for non-backbone peaks was unique to the assigned peptide, the non-backbone peaks were then shuffled between spectra of similar parent mass. The spectra were grouped into 100 bins of approximately 25 Da each. Non-backbone peaks were then swapped between spectra in the same bin. Although there are many duplicate spectra in the dataset, this resulted in non-backbone peaks being reassigned to spectra of the same peptide in< 1% of cases. Training PointNovo on these data resulted in a reduction in peptide recall of 7% when testing on unmodified real data (Supplementary Table 3). While this reduction in performance is much less than the reduction caused by removing all non-backbone peaks from the training data, it does suggest that some relationship exists between the assigned peptide and the non-backbone peaks.

Three different models of artificial non-backbone peaks were tested. These models were used to reintroduce the peaks back into the real data after they were removed. The performance of PointNovo was then compared using training data with non-backbone peaks removed versus training data with non-backbone peaks reintroduced artificially. Firstly, the non-backbone peaks were modelled as ions resulting from random peptide fragments since non-backbone peaks of unknown origin were found to be made up of amino acids (Fig. 5B). To create a non-backbone peak, a random number and selection of amino acids was sampled, with their combined mass defining the *m/z* value. The peak intensity was sampled from the distribution reported in our previous work [46]. The artificial addition of these peaks to the training data increased the peptide recall by 43% on real test data compared to training data with these peaks removed (Supplementary Table 3). Non-backbone peaks were also modelled solely as internal fragments as these also account for a large proportion of the non-backbone ions in tandem mass spectra (Fig. 1). Initially, all internal fragments were created for the matched peptide. Then a random sample of these were used to define the *m/z* values of the new peaks and the intensity values were sampled using the previously described distribution. Addition of these peaks to the training data with non-backbone peaks removed increased the peptide recall by 49% (Supplementary Table 3). Finally, non-backbone ions were introduced as a mixture of both random amino acids and internal fragments. Internal fragments were again sampled randomly but only enough to match their observed frequency. The remaining peaks were then added as random selections of amino acid sequences as before. Addition of non-backbone peaks using this combined method increased peptide recall by 58% compared to the training data with none present (Supplementary Table 3).

The presence or absence of a peak is related to its intensity as lower intensity ions are by definition less likely to appear in spectra. While the intensities of the peaks were found to be accurate, many peaks predicted by Prosit did not appear in real spectra. The addition to the training data of the peaks predicted by Prosit but absent from the real data caused an 11% reduction in peptide recall when tested on unmodified real data (Table 4).

The removal of *m/z* jitter associated with peptide peaks from the training data caused a 14% reduction in peptide recall (Table 4). As shown in Fig. 2, peaks do not appear exactly at the expected value. To remove the associated jitter, matched peaks were set to the expected *m/z* value. The *m/z* jitter was then reintroduced using two different distributions. The first is a mixture distribution of two normal distributions centered at zero with means of 1e-2 and 1e-3 in a 1:1 ratio. The reintroduction of this artificial jitter to the real training data resulted in a 6% increase in peptide recall (Supplementary Table 3). The jitter was also reintroduced using a mixture distribution of a Laplace distribution with a scale parameter of 2.5e-3 and a uniform distribution between − 0.05 and 0.05 with a 12:1 ratio. The introduction of this jitter resulted in a 5% increase in peptide recall (Supplementary Table 3).

**Fig. 6:**
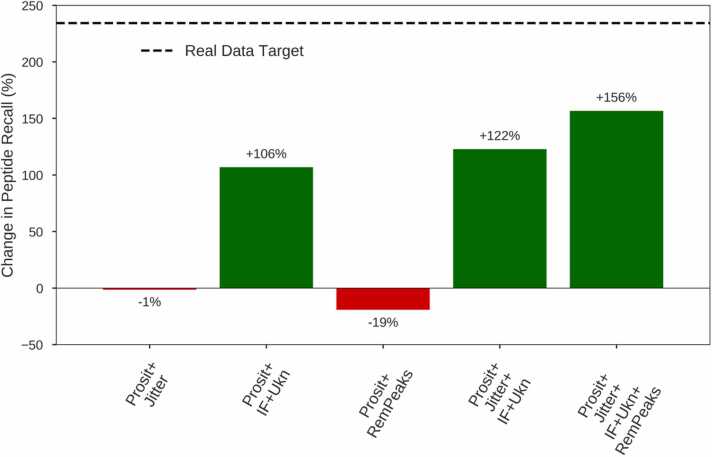
Change in performance of PointNovo [9] when trained on artificial spectra and tested on real spectra. The labels on the x-axis indicate the additions to the Prosit [29] generated training data. The real test spectra are from the yeast partition dataset, collated by Tran *et al.*[8]. Jitter signifies addition of *m/z* noise. IF indicates the addition of internal fragment noise peaks. Ukn indicates the addition of random peptide fragment noise peaks. RemPeaks indicates the removal of some of the lowest intensity peaks. The dashed line shows the performance of PointNovo trained on real spectra.

Using the understanding gained from modifying the real data by removing and replacing the different types of noise, the artificial spectra were then modified to improve their utility. The performance of PointNovo was assessed using real test data and modified artificial spectra as training data (Fig. 6).

Modifying the artificial training data with each of the different types of artificial noise improved the peptide recall by 156% compared to the unchanged artificial training data (Fig. 6). To put this in reference to real data, the peptide recall using artificial training data increased from 30% of the real training data value to 77% by adding these changes. Notably, not all of the introductions of variability improved performance when introduced alone. Both the introduction of jitter and the removal of peaks reduced performance by 1% and 19% respectively when they were the only changes to the artificial data. However, combined with the addition of internal fragments and unknown peaks they make significant improvements (Fig. 6).

Furthermore, it should be noted that PointNovo takes 12 ion types into account; a, b, y, a-H_2_O, b-H_2_O, y-H_2_O, a-NH_3_, b-NH_3_, y-NH_3_, a(2+), b(2+) and y(2+) ions. Only 4 of these overlap with the ion types predicted by Prosit; b, y, b(2+) and y(2+) ions. The peptide recall reached using artificial spectra in Fig. 6 is over 3/4 of the peptide recall reached when using real spectra, despite only using 1/3 of the fragment types. Further extension of the ion types predicted by Prosit would likely yield an increase in performance.

## 4 Discussion

Artificial data have been used extensively in machine learning to both train and test different models. In the context of *de novo* peptide identification it has only been used in the evaluation of models [15], [30], [31]. However, how applicable this approach is to real data performance has never been fully analysed.

In this research, PointNovo was first trained and tested on different combinations of unmodified real and artificial data. Testing on artificial data compared to real data testing reported greatly inflated performance. The artificial spectra had more peaks corresponding to fragment ions as well as no noise when compared to real spectra. This meant that the artificial spectra were much easier to classify than real spectra. Conversely, when training on artificial data the model began to overfit as the data had a much lower level of complexity. Testing this model on real data showed markedly decreased performance as the model was unable to deal with the noise of real spectra. It is evident from these tests that artificial spectra do not currently replicate the true complexity of real spectra.

To quantify these differences, the peaks attributable to the database assigned peptide in real tandem MS spectra were first classified. Previous studies have also aimed to categorise these peaks [48], [60]. However, the work presented here describes different data from a different mass spectrometer. Furthermore, random peptides were used to estimate the number of spuriously matched peaks and include quantification of the different forms of noise associated with real spectra. The real data were also compared to artificial spectra created using the Prosit pipeline.

By matching the peaks to fragment ions attributable to the assigned peptides from a database search, 36% of peaks in the spectra were accounted for (Fig. 1). However, 13% of peaks in the spectra were also matched to fragment ions attributable to a random peptide (Fig. 1). Random peptides were used in this research to provide an estimate of the number of spurious fragment ion matches. Taking this into consideration, 77% of the peaks in the spectra could not be attributed to the database assigned peptides.

The creation of random peptides also allowed us to estimate the number of spurious matches for each ion type. This should guide informed selection of ion types that should be included in future *de novo* models. If an ion does not appear very often and is likely to be matched by chance, it may not be very useful for peptide prediction. While an ion type that appears more often than chance can still provide additional information toward the peptide prediction process, it must be balanced, however, with a trade-off in model complexity. Indeed, the inclusion of each additional ion type increases the model complexity, making training and prediction slower, as well as requiring more computational resources.

Internal fragments are not currently utilised in *de novo* peptide identification algorithms. However, 60% of all possible internal fragments of length 2 were matched in the spectra. While further research is required to assess how this might be realised, the inclusion of internal fragments could be explored in future *de novo* algorithms. Previous work highlighted the need for complete spectrum encoding to counteract missing fragmentation cleavages [15], a strategy that would inherently include internal fragments into the prediction process. A recent approach to *de novo* peptide identification using transformers, Casanovo, encodes every peak in the spectrum thereby also including internal fragments [61]. With this additional information Casanovo reported a mean improvement of 1.3% over PointNovo in peptide precision [61].

The creation of representative artificial data necessitates the inclusion of all peaks present in the spectra, not only backbone ions. Liu *et al.* created a model, PredFull, that attempted to completely recreate tandem MS spectra [62] using a bin size of 0.1 Da, which is large in light of the precision of modern mass spectrometers. The Q-exactive used to create the data for this research has a maximum precision of< 1 ppm [63]. Training PointNovo using PredFull spectra and testing on real spectra resulted in a peptide recall of 0.20. This was better than Prosit alone (0.15) but not better than the peptide recall achieved in this work through our modifications (0.38).

As artificial data are synthetic, they lack much of the noise associated with real data. In this research, four types of noise that only appear in real data were categorised; *m/z* jitter, missing peaks, intensity and unknown peaks. Despite being deterministic, Prosit was found to predict the intensity with a mean error of less than 0.05. Using this deterministic artificial intensity in the training data was found to have very little effect on model performance (1% reduction). Therefore the artificial intensity was not further modified. As there is such a large diversity of peptides in the training data, variability in intensity between spectra of the same peptide is of minimal importance.

Using a novel plot of *m/z* versus *m/z* modulo 1, this work was able to provide evidence as to the origin of the unknown peaks in MS/MS spectra. The mean *m/z* to nucleon ratio of the unknown peaks was found to be indicative of molecules made of amino acids. This suggests that the unknown peaks in tandem MS spectra are due to peptide contaminants or coeluting peptides. This information was then used to create a model to recreate these peaks in artificial spectra.

Finally, the models of three different types of noise were combined with the Prosit predicted spectra. This increased the peptide recall of a model trained on artificial spectra from 30% to 77% of the peptide recall of a model trained on real spectra. While substantial improvements are still required to realise the full potential of artificial data in the context of *de novo* peptide identification, this work has made significant progress in this area.

Continued improvements would transform artificial spectra into a tool for the systematic and comprehensive analysis of *de novo* algorithms. Artificial data can facilitate evaluation at adjustable levels of data complexity such as increased noise. Observing how the performance of a model is impacted by changes in specific data characteristics would provide valuable insights into its strengths and weaknesses. Currently, models must be tested on real spectra where the effects of different data characteristics are difficult to separate [15]. Furthermore, supplementing the training data with these modified spectra may also help reduce the bias associated with database labelled spectra. While artificial spectra may not completely replace real training data, they can provide diverse or rare examples to the model to help improve performance. Difficult to classify spectra will by definition appear less often in the training data obtained using a database search. This will in turn make it difficult for *de novo* models to learn to classify such spectra. Also, as *de novo* performance is always benchmarked using this subset of high-scoring spectra, the extent of this issue is not easy to quantify. The artificial generation of difficult-to-classify spectra, those with many missing fragmentation cleavages [15], may offer both a way to identify this bias as well as alleviate it through supplementation of the training data. A problem to note here is that models used to generate artificial spectra also currently rely on database labelled spectra as training data. These models will therefore likely have their own bias. Further research is needed to see if the addition of noise will diversify these artificially generated spectra enough to mitigate against their own inherent bias when used as training or test data for *de novo* peptide identification models.

## 5 Conclusion

This work provides a critical analysis of artificial data in the context of *de novo* peptide identification algorithms. It presents a comprehensive survey of the different peptide fragment ions matched in tandem MS spectra and provides evidence for the origins of unmatched peaks. While this research shows the current limitations of using artificial data to train or evaluate *de novo* peptide identification algorithms, it also highlights its future potential. The inclusion of additional noise was shown to significantly increase the utility of artificial spectra for model training. High quality artificial spectra could help alleviate the reliance of current algorithms on a database search for generating training data. Furthermore, such artificial spectra could allow for the quantification of the effects of specific data characteristics on *de novo* peptide identification. A greater understanding of the challenges facing *de novo* algorithms is necessary to he design of more robust future models. This work represents the first step toward such a new approach to *de novo* peptide identification model training and evaluation.

## CRediT authorship contribution statement

**Kevin McDonnell**: Conceptualisation, Methodology, Writing – original draft preparation. **Enda Howley**: Conceptualisation, Writing – reviewing and editing. **Florence Abram**: Conceptualisation, Writing – reviewing and editing.

## Declaration of Competing Interest

The authors declare that they have no known competing financial interests or personal relationships that could have appeared to influence the work reported in this paper.
