# Supplementary material for: Critical evaluation of the use of artificial data for machine learning based de novo peptide identification
Source: Comput Struct Biotechnol J. 2023 Apr 17;21:2732–43. doi: 10.1016/j.csbj.2023.04.014 (PMC10165132; doi:10.1016/j.csbj.2023.04.014)
Supplement: Supplementary file 1 — Supplementary material [file mmc1.pdf]

Supplementary Data

Critical evaluation of the use of artificial data for machine learning based *de novo* peptide identification

Kevin McDonnell[a,b,*], Enda Howley[b] and Florence Abram[a]

[a] Functional Environmental Microbiology, School of Natural Sciences, Ryan Institute, University of Galway, Ireland
[b] Department of Information Technology, School of Computer Science, University of Galway
[*] kevinmcdonnell.ug@gmail.com
florence.abram@universityofgalway.ie

1 Estimating Random Matches

Alongside the method used to estimate the number of random matches in the main manuscript, we also investigated several others. The first of these involved the generation of a random peptide from amino acids not present in the database assigned peptide but of the same length (R_NoShare). This generates highly unlikely, non-tryptic peptides which give a lower bound estimate of how often fragment ions are assigned by chance. This is shown by the relatively lower values observed in Supplementary Table 1.

The second method was the scrambling of database assigned peptide but maintaining the last amino acid (R_Scramble). This is a method used in the generation of decoy databases and maintains the same amino acid composition as the original set of peptides. With the same amino acids used, this may lead to an overestimation of the randomly matched internal fragments. Indeed this method matched the largest number of internal fragments of those used (Supplementary Table 1). With many ions shared with the original peptide, this likely gives an upper bound to the number of randomly matched ions.

Finally, we also randomly shuffled the spectra while maintaining the original peptides to estimate the spurious matches (R_Spectrum). This method has the advantage of searching for the same theoretical fragment ions as the original search. Database assigned peptides were compared to the spectra of peptides of similar mass ($\Delta m < 25$Da) so that the fragment ions spanned the same m/z range as the observed ions.

Randomly generated tryptic peptides had a larger relative amount of y ion matches than non-tryptic peptides, even though the last amino acid in the sequences were different to those of the assigned peptides. This is partly explained by the frequency with which both arginine and lysine y_1 ions are matched in the spectra (Supplementary Table 2). Despite the assigned peptides only ending with in R or K (excluding rare exceptions), y_1 ions for both amino acids were present in almost all spectra. Similar to Figure 5 in the main manuscript, this would indicate that more peptides are present in the spectra than those detected by the database search.

Ion Type	#R_NoShare	#R_NoShare / #Matched	#R_Scramble	#R_Scramble / #Matched	#R_Spectrum	#R_Spectrum / #Matched
Backbone	45373	5%	169940	18%	128288	14%
a	20765	13%	47786	31%	39176	25%
b	13146	5%	45971	17%	32020	12%
y	11462	2%	76183	15%	57092	11%
Charge 2+	24405	15%	103154	64%	84001	52%
a(2+)	9379	22%	22438	52%	20312	47%
b(2+)	7444	15%	18763	37%	17468	34%
y(2+)	7582	11%	24524	37%	22536	34%
Ion Loss	27124	5%	65725	13%	60316	12%
a-H20	1047	4%	8782	33%	6876	26%
b-H20	573	1%	7348	14%	4837	9%
y-H20	12580	6%	56065	26%	45880	21%
a-NH3	5436	14%	10324	26%	9647	24%
b-NH3	3465	5%	7694	12%	6839	10%
y-NH3	4023	4%	12941	13%	9923	10%
Int Frags	626938	38%	1075432	65%	957554	58%
b	274051	27%	562521	55%	485171	47%
a	352887	56%	512911	81%	472383	75%

Table 1: Estimates of the number of randomly matched peaks of different ion types in a sample of 50,000 HCD PSMs with a matching tolerance of 0.05 Da. The data are from 9 different organisms and research groups, collated by Tran *et al.*. Columns indicate the number of ions from each method that were matched (#R_Type), and the ratio of the number of ions matched from the random peptides to the number of ions matched from the assigned peptides (#R_Type/#Matched). R_NoShare: Random sample of amino acids not present in assigned peptide, R_Scramble: Assigned peptides are scrambled while keeping the same last amino acid, R_Spectrum: Assigned peptides are compared to randomly selected spectra.

	K	R
Last AA in Assigned Peptide	28948	19557
y_1 Ion Matched in Spectrum	47957	45928

Table 2: The number of arginine and lysine y_1 fragments matched in a sample of 50,000 HCD PSMs with a matching tolerance of 0.05 Da. The data are from 9 different organisms and research groups, collated by Tran *et al.*.

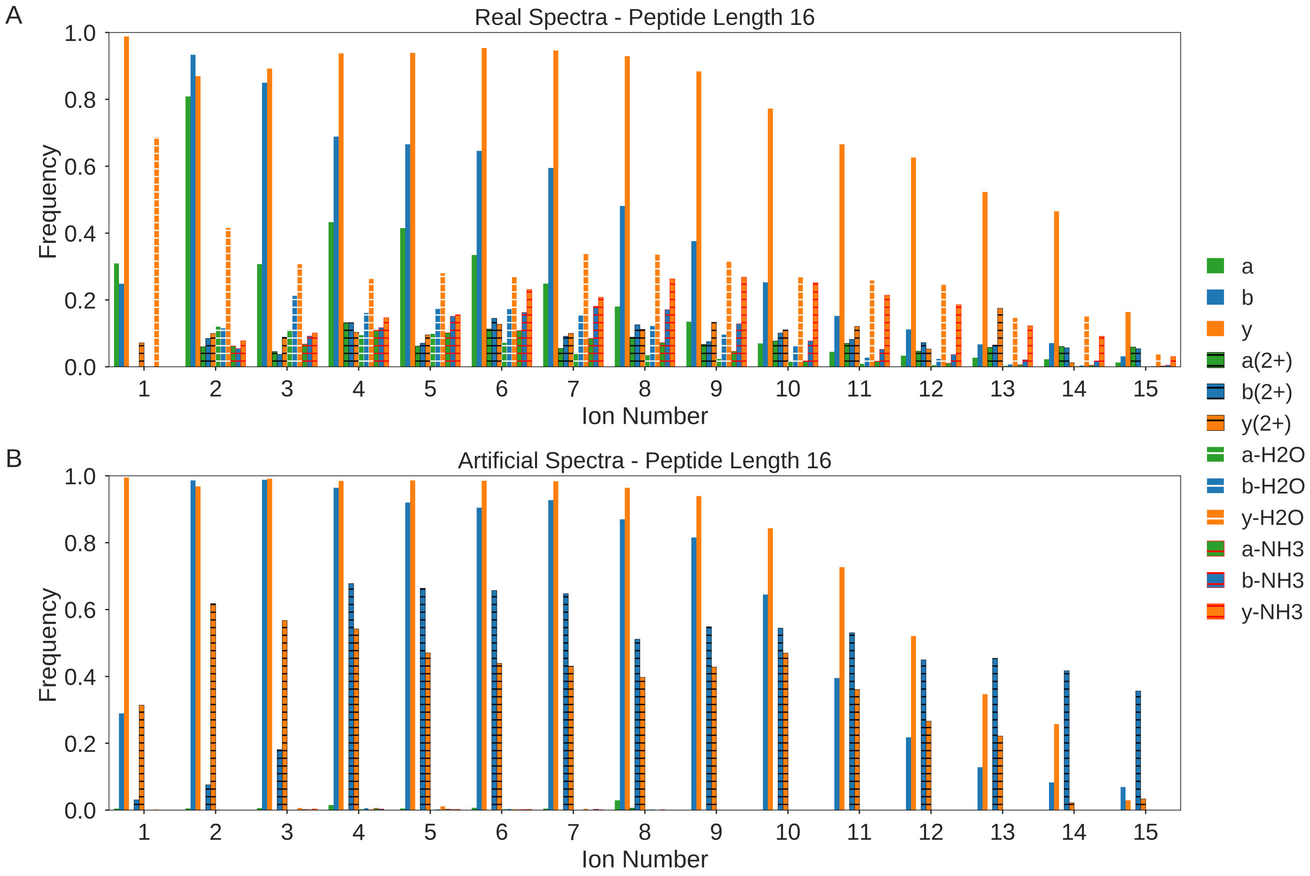

Supplementary Figure 1: Distribution of the presence of 12 different ion types in real and artificial spectra for length 16 peptides. Ions of the same type share the same base colour with different colour hatching indicating different charge states or neutral losses.

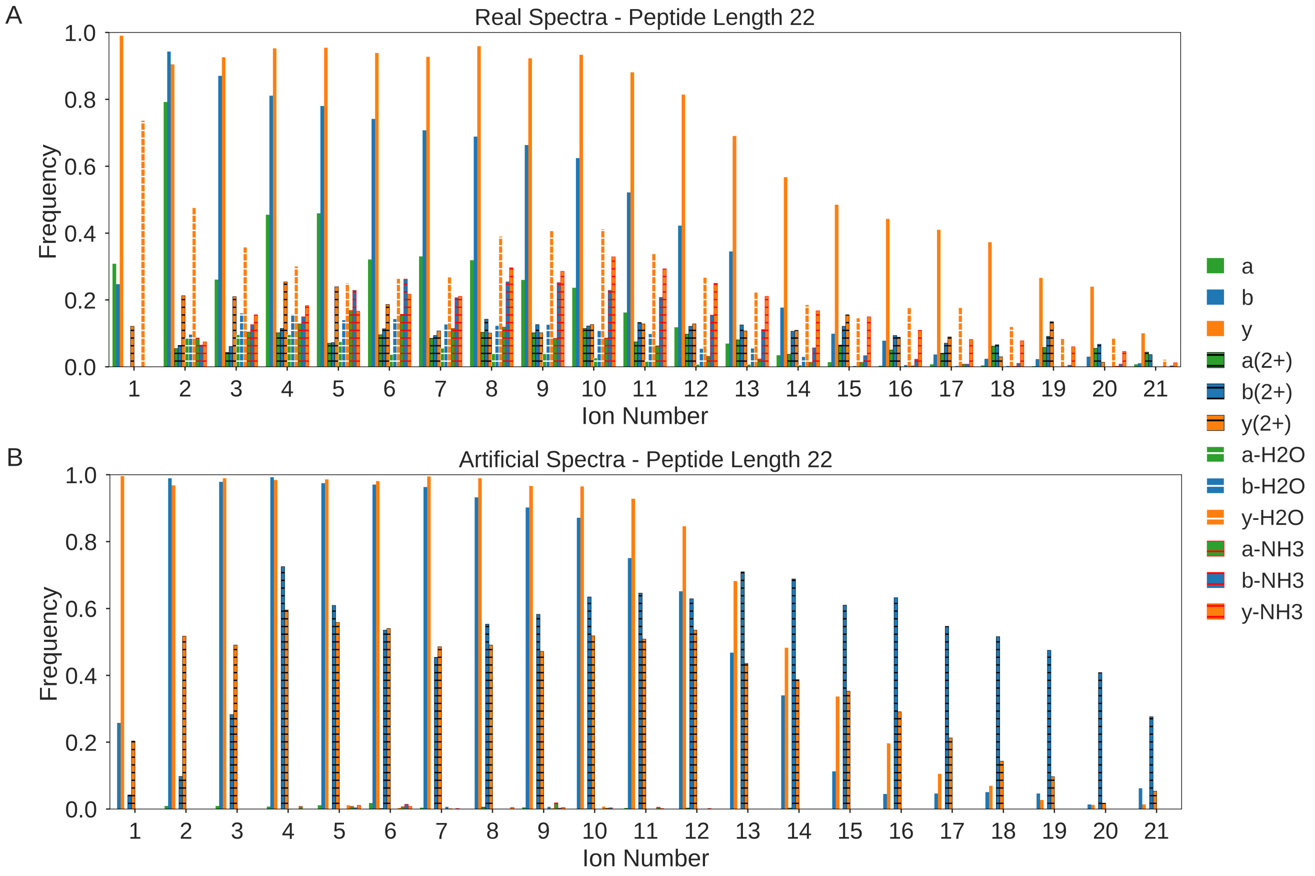

Supplementary Figure 2: Distribution of the presence of 12 different ion types in real and artificial spectra for length 22 peptides. Ions of the same type share the same base colour with different colour hatching indicating different charge states or neutral losses.

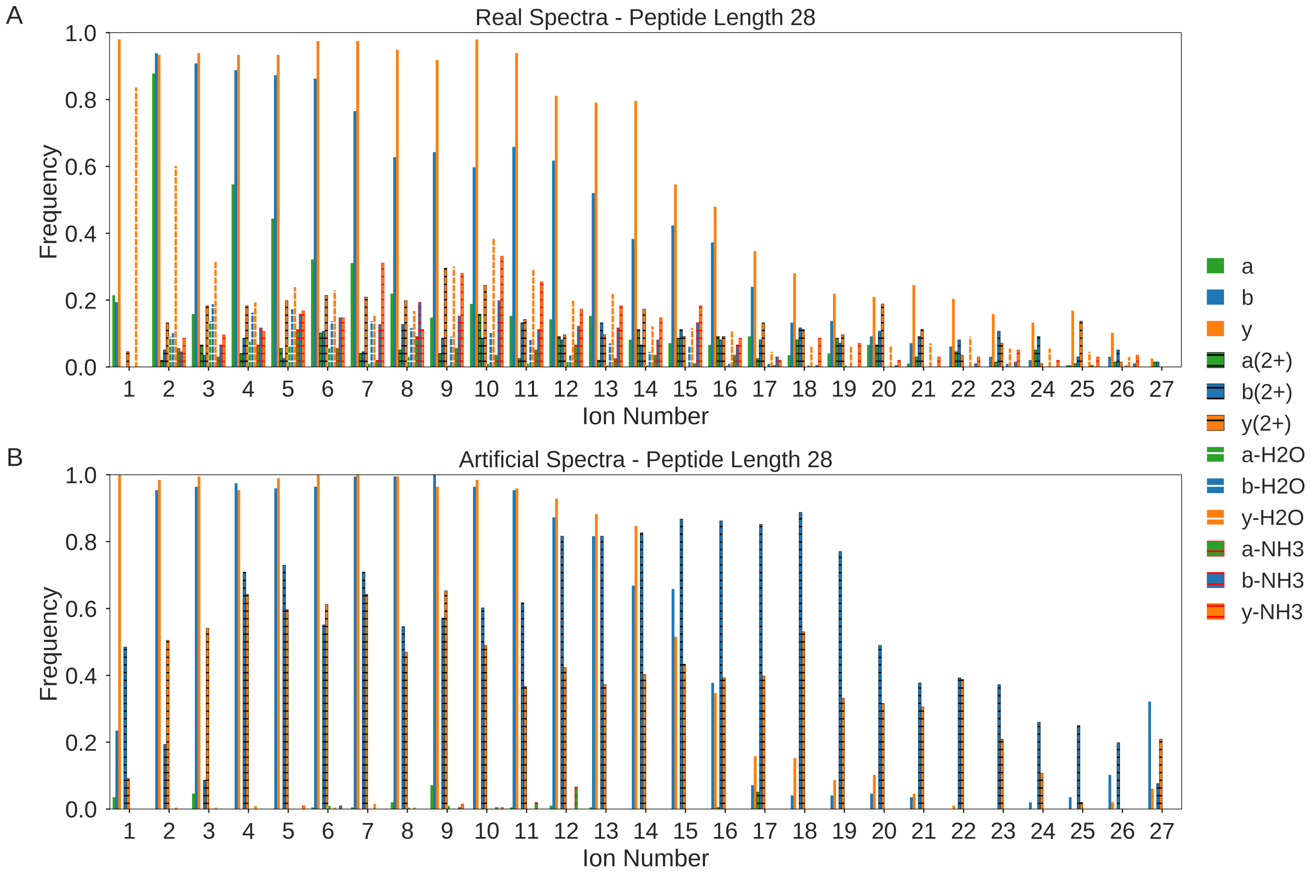

Supplementary Figure 3: Distribution of the presence of 12 different ion types in real and artificial spectra for length 28 peptides. Ions of the same type share the same base colour with different colour hatching indicating different charge states or neutral losses.

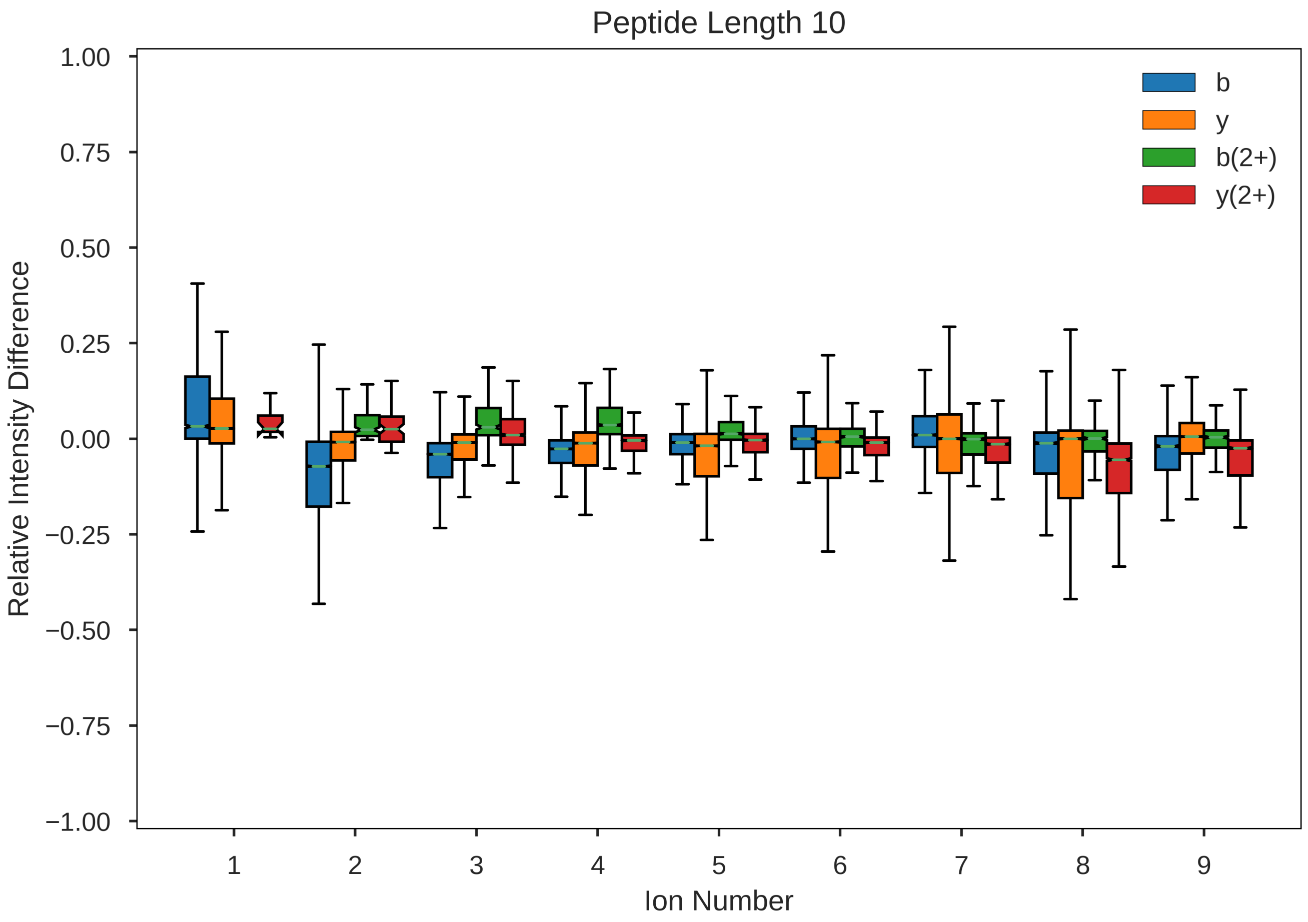

Supplementary Figure 4: Distribution of the difference in relative intensity predicted by Prosit and the observed value for length 10 peptides. All real intensities are normalised to the maximum fragment ion intensity matched.

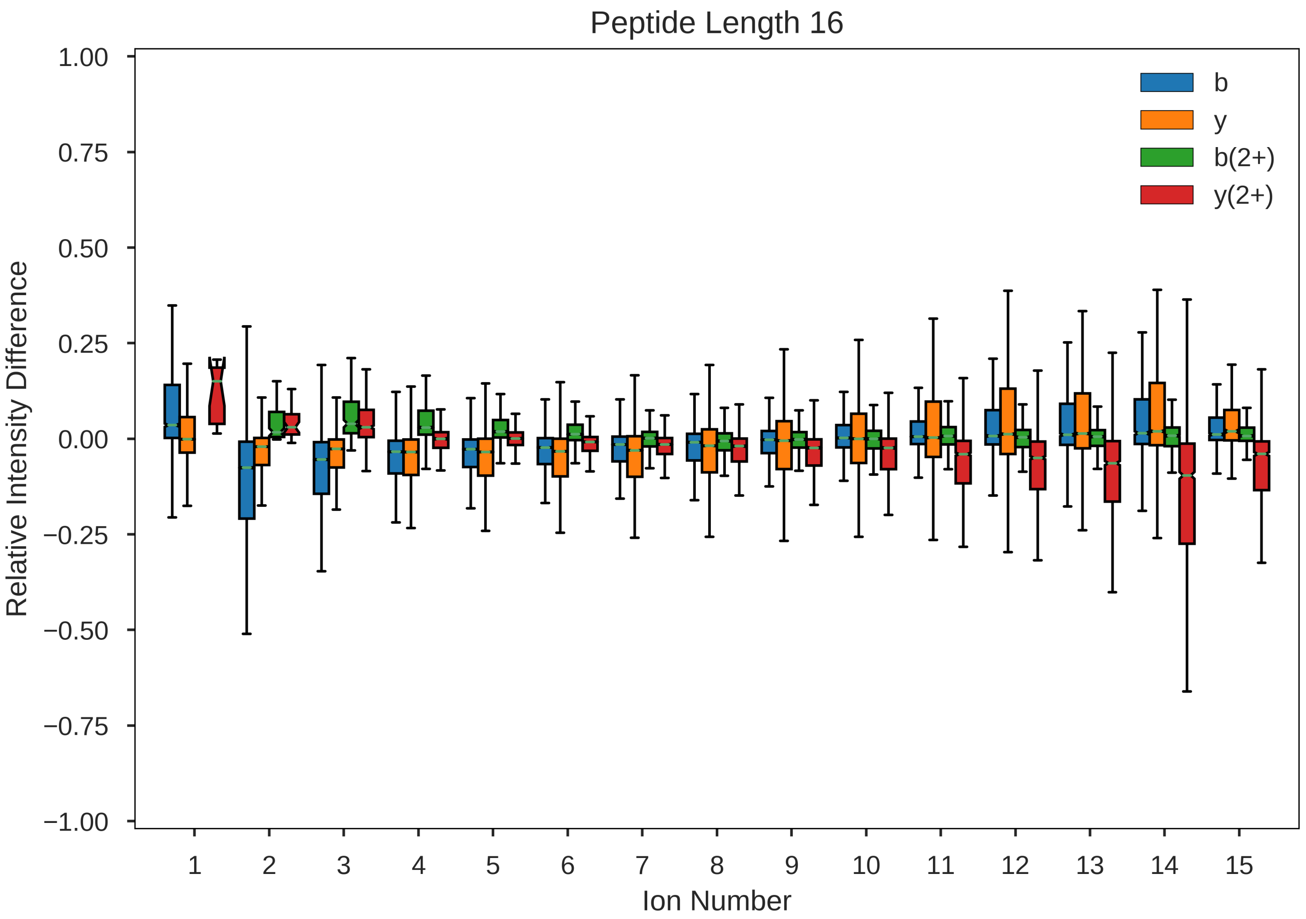

Supplementary Figure 5: Distribution of the difference in relative intensity predicted by Prosit and the observed value for length 16 peptides. All real intensities are normalised to the maximum fragment ion intensity matched.

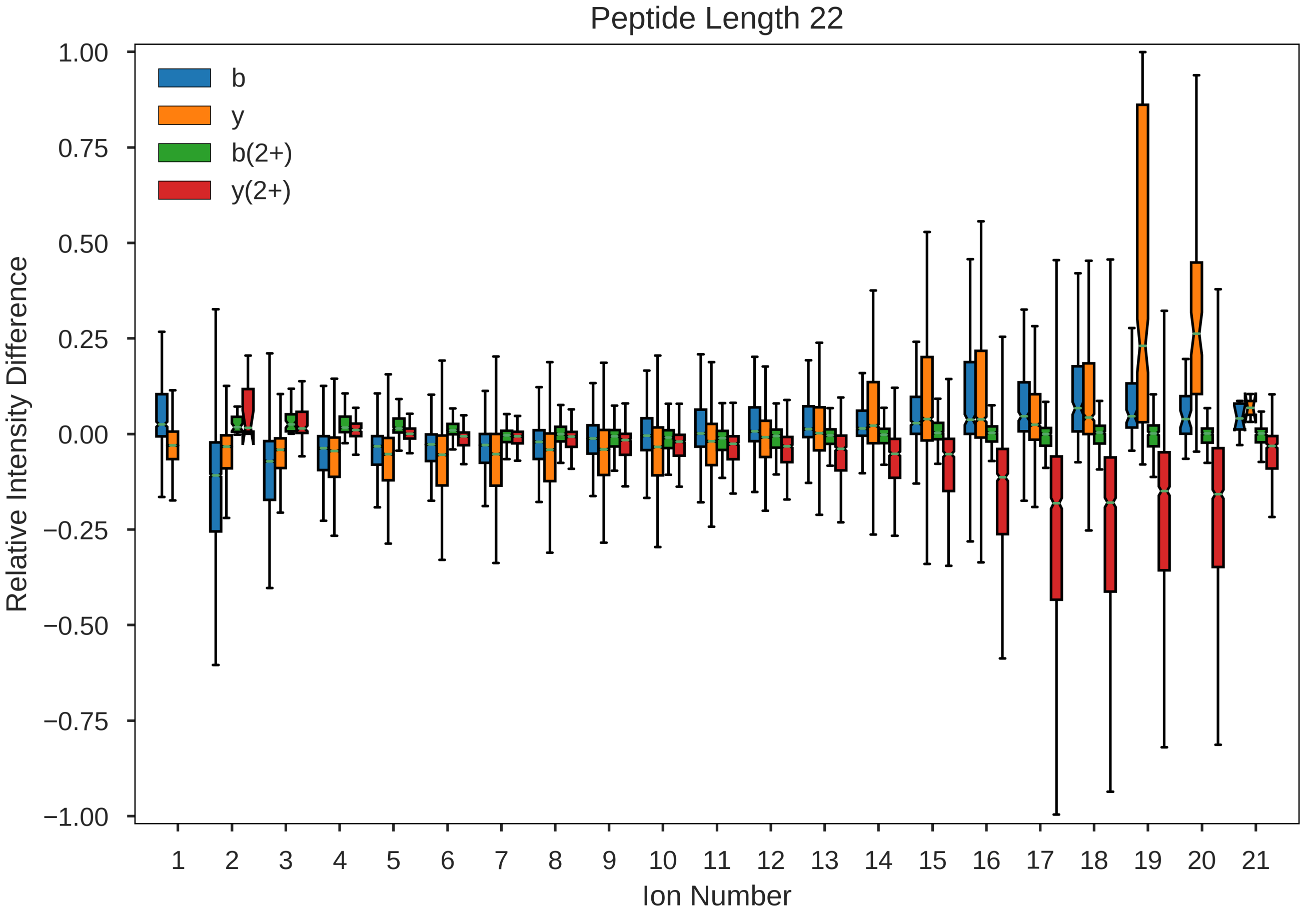

Supplementary Figure 6: Distribution of the difference in relative intensity predicted by Prosit and the observed value for length 22 peptides. All real intensities are normalised to the maximum fragment ion intensity matched.

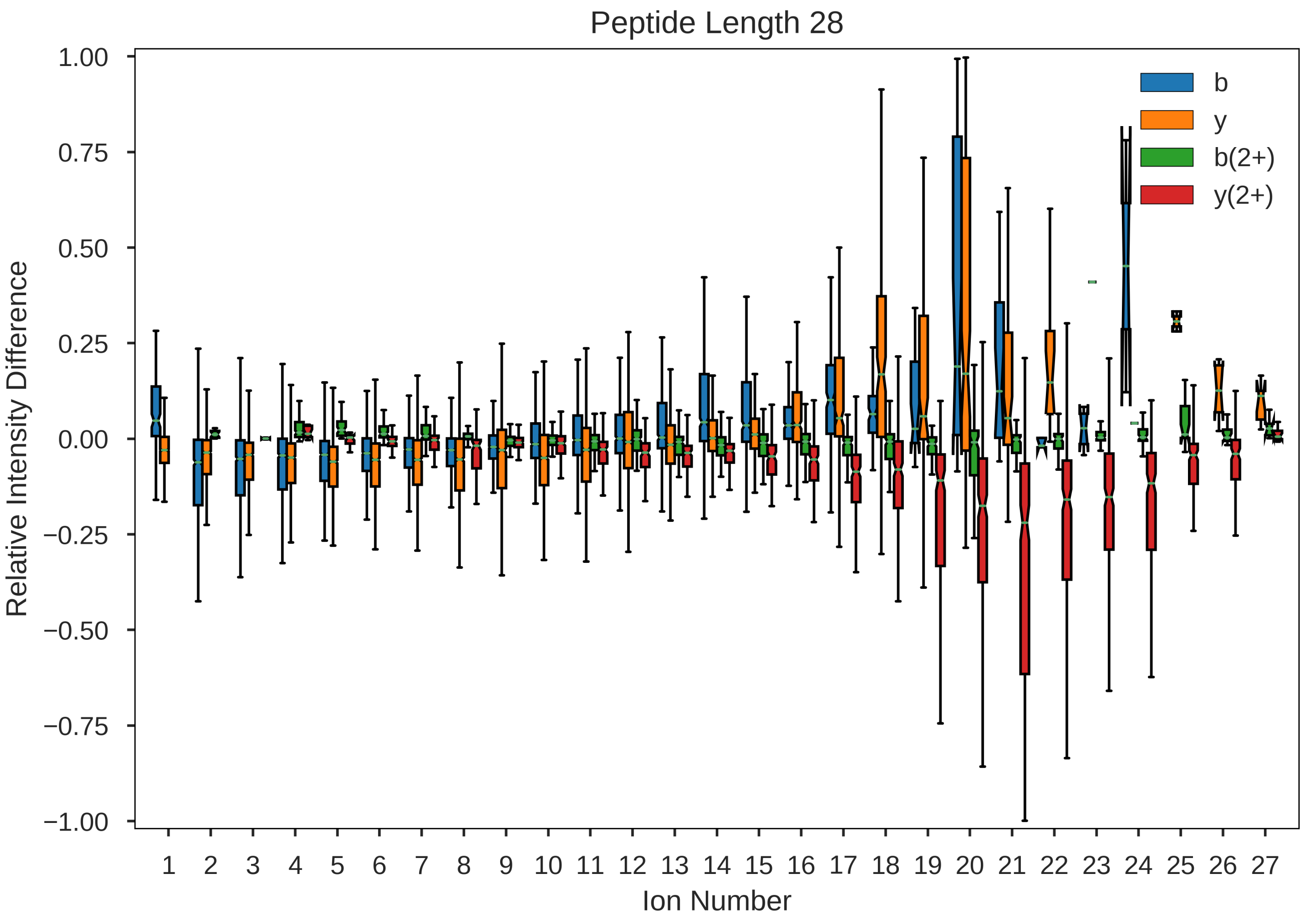

Supplementary Figure 7: Distribution of the difference in relative intensity predicted by Prosit and the observed value for length 28 peptides. All real intensities are normalised to the maximum fragment ion intensity matched.

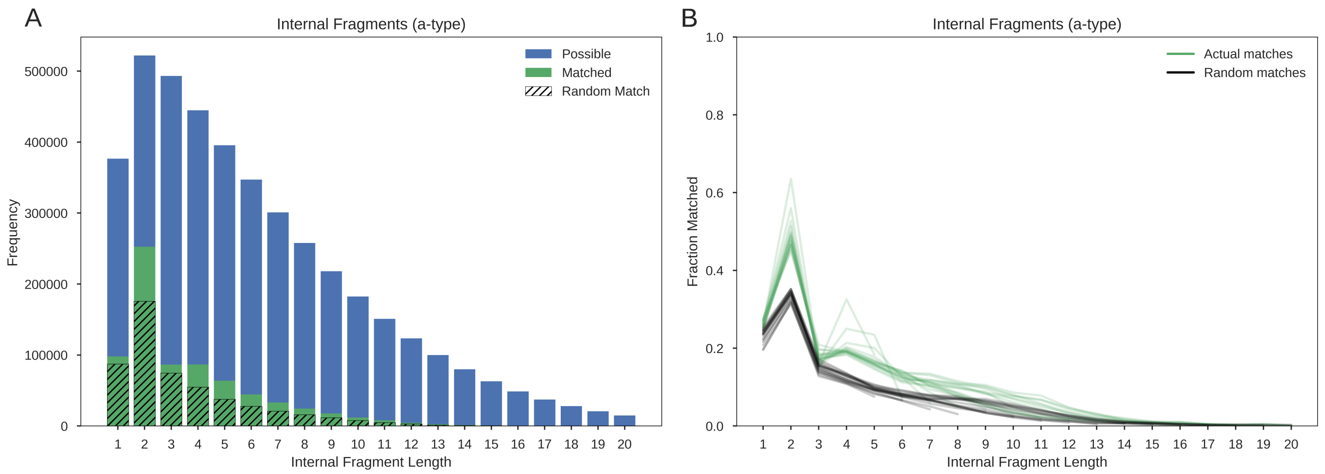

Supplementary Figure 8: The number of a-type internal fragments matched by length. A shows the counts of possible unique internal fragment masses (blue), matched internal masses (green), matched random internal masses (black hatch). B shows the fraction of the total number of possible internal fragments matched by the actual peptides (green) and the random peptides (black). Each individual line represents the different peptide lengths.

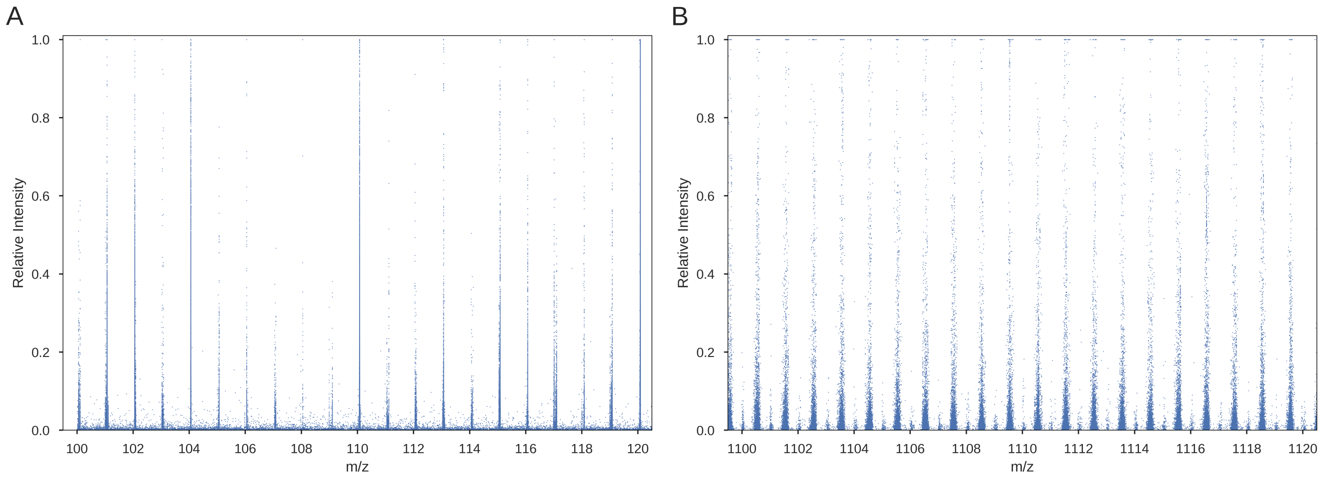

Supplementary Figure 9: Distribution of m/z values vs relative intensity values for peaks in a sample of 50,000 spectra. A shows peaks with m/z values between 100 and 120. B shows peaks with m/z values between 1100 and 1120.

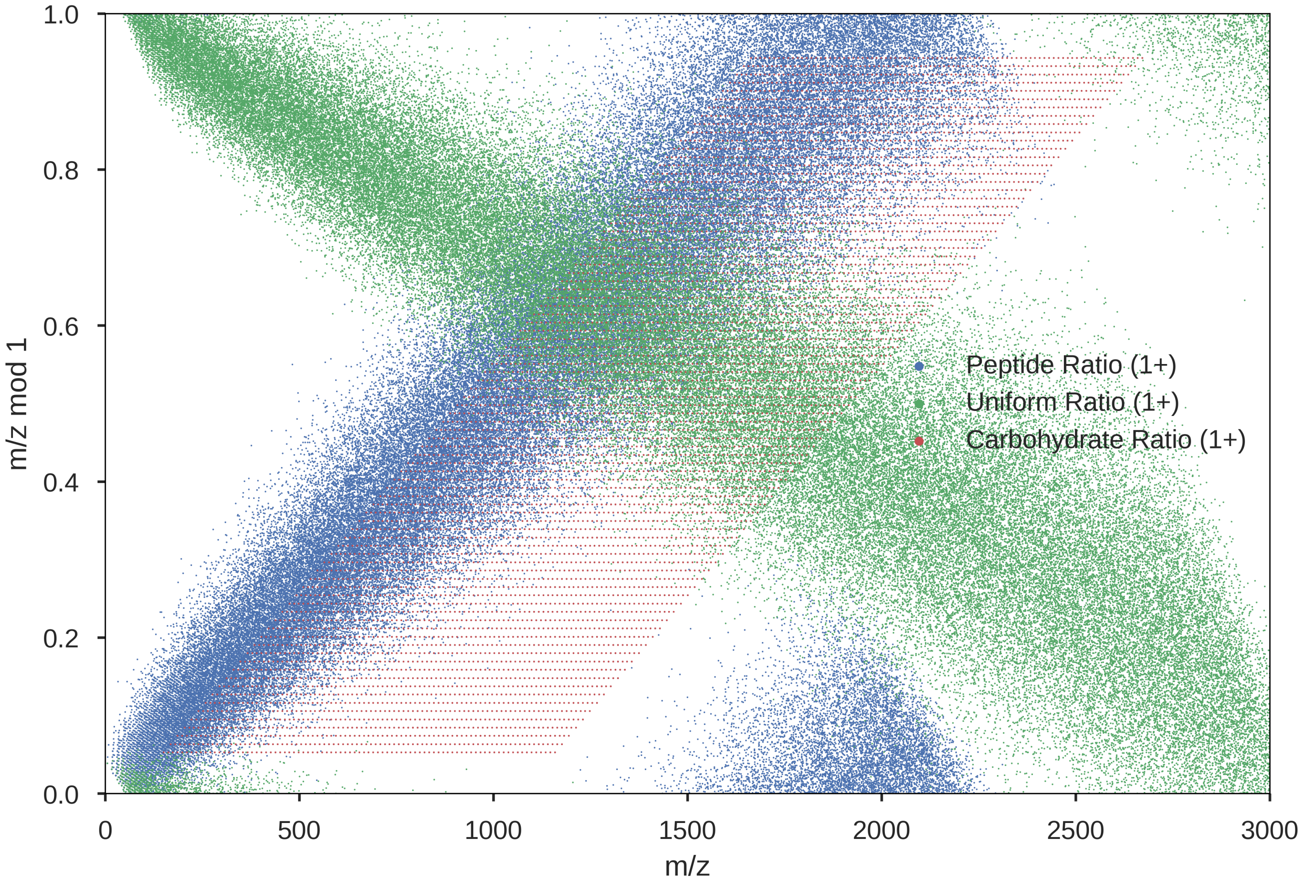

Supplementary Figure 10: Distribution of m/z values vs m/z modulo 1 for molecules with different ratios of hydrogen, carbon, nitrogen, oxygen and sulphur.

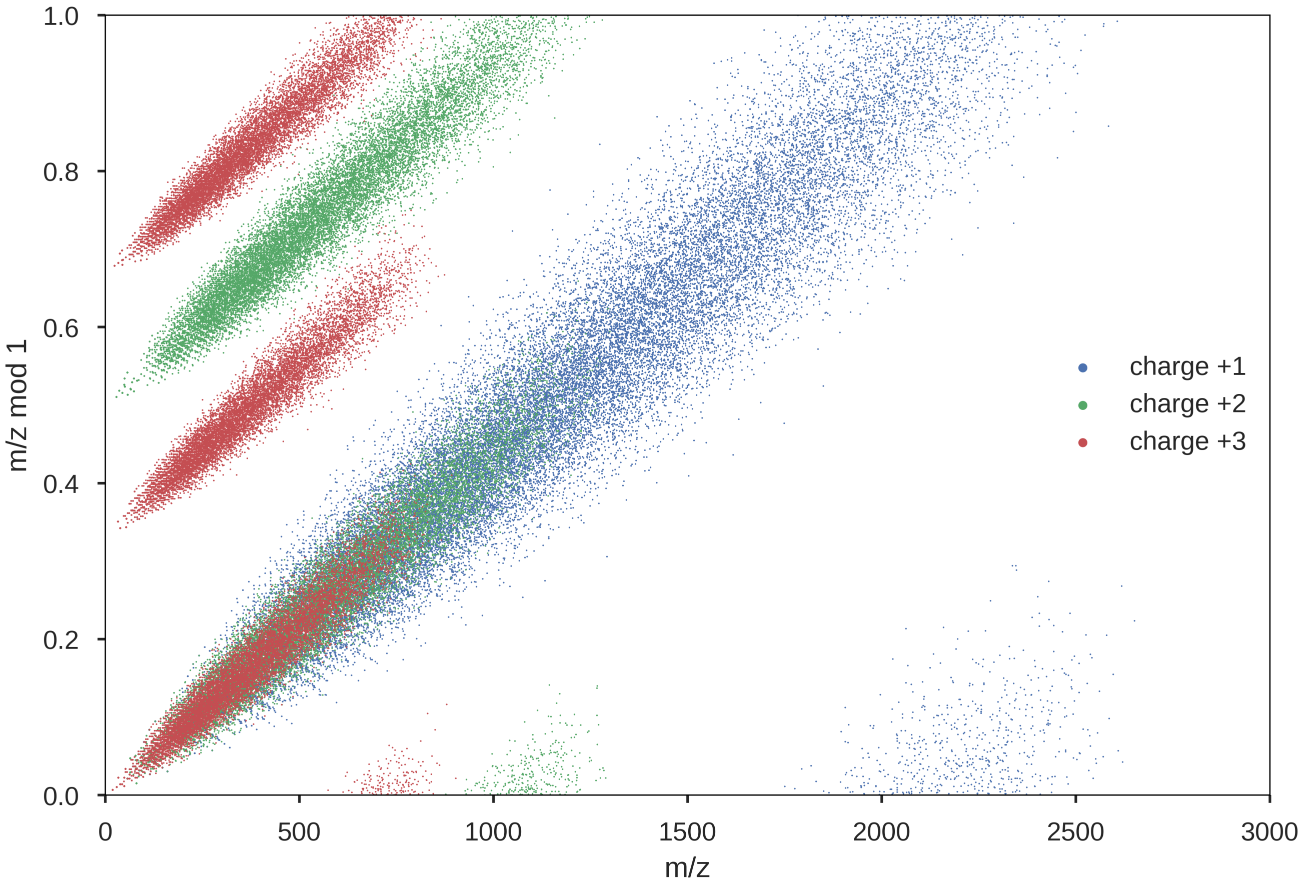

Supplementary Figure 11: Distribution of m/z values vs m/z modulo 1 for random peptide fragment peaks of different charges.

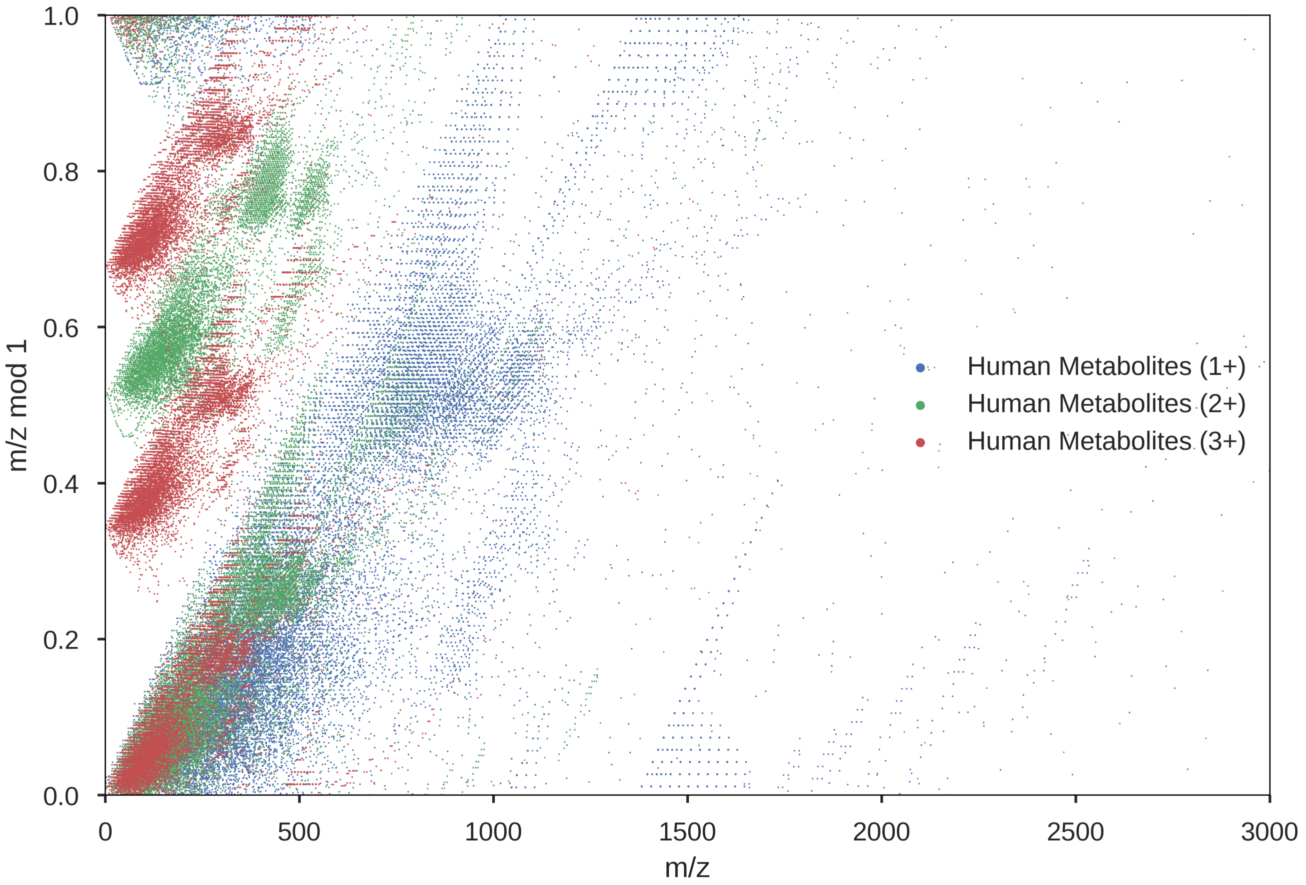

Supplementary Figure 12: Distribution of m/z values vs m/z modulo 1 for human metabolites of different charges.

Modification Type	AA Recall	AA Precision	Peptide Recall
Add Normal Jitter m/z	0.6820	0.6813	0.4507
Add Exp. Jitter m/z	0.6803	0.6799	0.4499
Shuffle Non-BB Peaks	0.6987	0.6989	0.4616
Add Random AA Peaks	0.5920	0.5911	0.3511
Add Random IF peaks	0.5909	0.5886	0.3646
Add Random IF+AA peaks	0.6082	0.6101	0.3881

Table 3: Performance of PointNovo when trained using modified real spectra. The training data had noise either shuffled between spectra or removed and reintroduced artificially using the specified method.
